# Web platform using digital image processing and geographic information system tools: a Brazilian case study on dengue

**DOI:** 10.1186/s12938-015-0052-2

**Published:** 2015-07-16

**Authors:** Lourdes M Brasil, Marília M F Gomes, Cristiano J Miosso, Marlete M da Silva, Georges D Amvame-Nze

**Affiliations:** Biomedical Engineering Graduate Program, University of Brasília at Gama, Área Esp. 2 Lote 14 Setor Central, Gama, Brasília, Brazil

**Keywords:** Dengue, Ovitraps, Image processing, Geographic information system

## Abstract

**Background:**

Dengue fever is endemic in Asia, the Americas, the East of the Mediterranean and the Western Pacific. According to the World Health Organization, it is one of the diseases of greatest impact on health, affecting millions of people each year worldwide. A fast detection of increases in populations of the transmitting vector, the *Aedes aegypti* mosquito, is essential to avoid dengue outbreaks. Unfortunately, in several countries, such as Brazil, the current methods for detecting populations changes and disseminating this information are too slow to allow efficient allocation of resources to fight outbreaks. To reduce the delay in providing the information regarding *A. aegypti* population changes, we propose, develop, and evaluate a system for counting the eggs found in special traps and to provide the collected data using a web structure with geographical location resources.

**Methods:**

One of the most useful tools for the detection and surveillance of arthropods is the ovitrap, a special trap built to collect the mosquito eggs. This allows for an egg counting process, which is still usually performed manually, in countries such as Brazil. We implement and evaluate a novel system for automatically counting the eggs found in the ovitraps’ cardboards. The system we propose is based on digital image processing (DIP) techniques, as well as a Web based Semi-Automatic Counting System (SCSA-WEB). All data collected are geographically referenced in a geographic information system (GIS) and made available on a Web platform. The work was developed in Gama’s administrative region, in Brasília/Brazil, with the aid of the Environmental Surveillance Directory (DIVAL-Gama) and Brasília’s Board of Health (SSDF), in partnership with the University of Brasília (UnB). The system was built based on a field survey carried out during three months and provided by health professionals. These professionals provided 84 cardboards from 84 ovitraps, sized 15 × 5 cm. In developing the system, we conducted the following steps:

i. Obtain images from the eggs on an ovitrap’s cardboards, with a microscope.
ii. Apply a proposed image-processing-based semi-automatic counting system. The system we developed uses the Java programming language and the Java Server Faces technology. This is a framework suite for web applications development. This approach will allow a simple migration to any Operating System platform and future applications on mobile devices.
iii. Collect and store all data into a Database (DB) and then georeference them in a GIS. The Database Management System used to develop the DB is based on PostgreSQL. The GIS will assist in the visualization and spatial analysis of digital maps, allowing the location of Dengue outbreaks in the region of study. This will also facilitate the planning, analysis, and evaluation of temporal and spatial epidemiology, as required by the Brazilian Health Care Control Center.
iv. Deploy the SCSA-WEB, DB and GIS on a single Web platform.

**Results:**

The statistical results obtained by DIP were satisfactory when compared with the SCSA-WEB’s semi-automated eggs count. The results also indicate that the time spent in manual counting has being considerably reduced when using our fully automated DIP algorithm and semi-automated SCSA-WEB. The developed georeferencing Web platform proves to be of great support for future visualization with statistical and trace analysis of the disease.

**Conclusions:**

The analyses suggest the efficiency of our algorithm for automatic eggs counting, in terms of expediting the work of the laboratory technician, reducing considerably its time and error counting rates. We believe that this kind of integrated platform and tools can simplify the decision making process of the Brazilian Health Care Control Center.

## Background

We propose digital image processing (DIP) and geographic information system (GIS) methods and tools to retrieve information about the *Aedes aegypti* eggs, as extracted from ovitraps. This work is based on an original project implemented at the University of Brasília (UnB), called Acquisition and Processing System of Ovitraps Images (SAPIO, from the Portuguese form *Sistema de Aquisição e Processamento de Imagens de Ovitrampas*). This project was also supported by the Federal University of Pernambuco (UFPE) [1].

Dengue fever is endemic in Asia, the Americas, the East of the Mediterranean and Western Pacific. It is one of the diseases of greatest impact on health, affecting more than 100 million people a year worldwide, according to the World Health Organization [2].

The global distribution of dengue is comparable to that of malaria, and there is an estimated population of 2.5 billion people living in areas of potential risk. The disease affects 50 to 100 million people annually, leading to 24 thousand deaths and a number from 250,000 to 500,000 severe cases known as dengue hemorrhagic fever (DHF). Dengue has lead to high mortality rates in recent decades, particularly in children, and the rate of fatal cases in most countries is 5% [3].

Dengue is associated to the most important arbovirus to affect mankind nowadays, and it constitutes a serious public health problem in the world, especially in tropical countries [3]. In these locations, the environmental conditions favors the development and proliferation of the *A. aegypti*, and the prevention and treatment of DHF is usually very high, with at least 70% of cases occurring from January to May [4].

**Figure 1:**
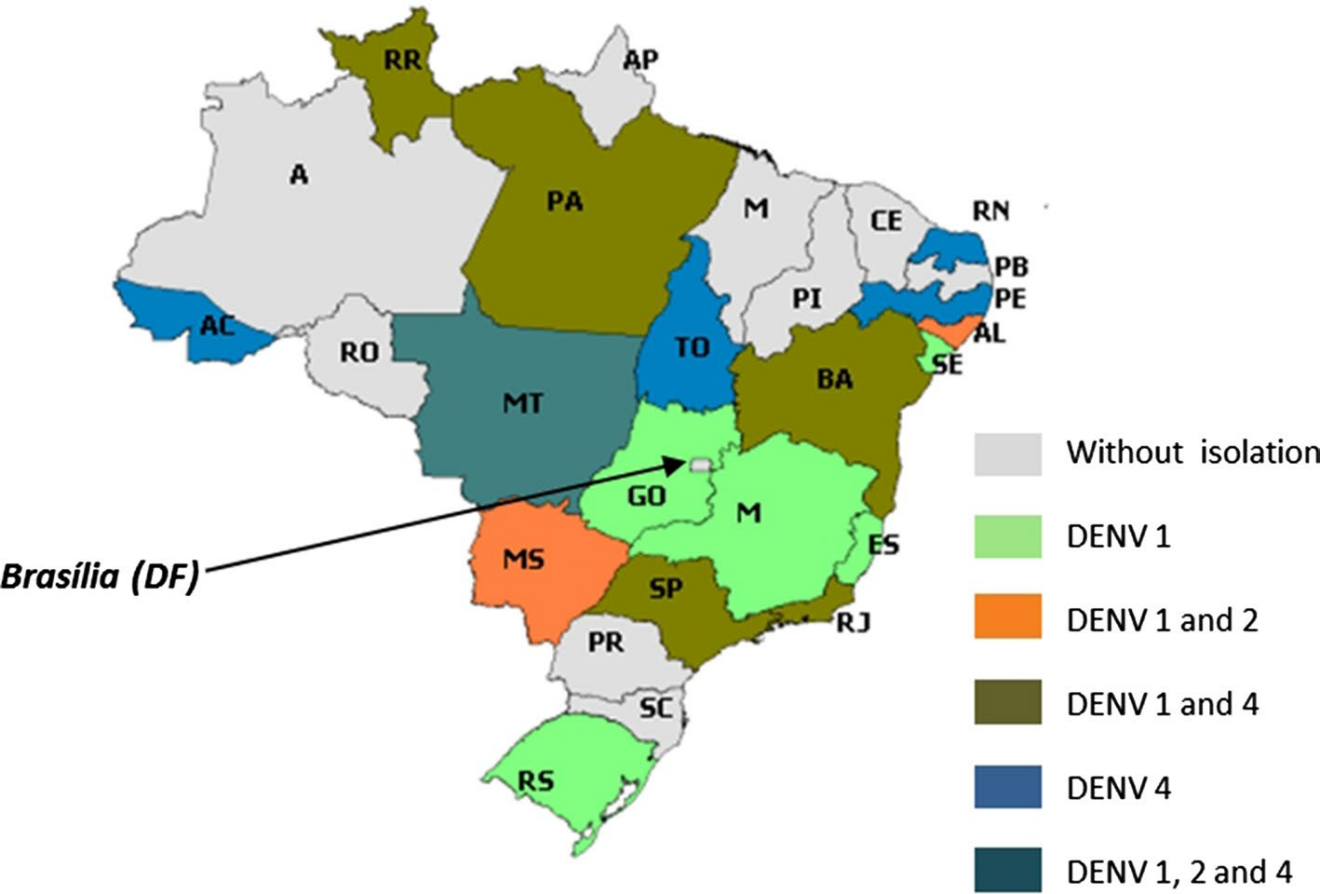
Brazilian estimate of dengue fever serotypes in 2012 [4].

Brazil, for example, has four types of dengue fever serotypes: DENV-1, DENV-2, DENV-3 and DENV-4, as Figure 1 illustrates. The newly discovered of serotype DENV-5 [5] has not yet been reported in Brazil.

The control of this disease, typically urban and multi sectoral, is quite complex and involves: health public programs, cities infrastructure, people and materials logistics, environment and education, among others. Thus, this disease generates high cost in terms of hospital expenses, prevention programs, surveillance, vectors control and mobilization of the population. In this sense, it is necessary to explore new alternatives to assist and facilitate the control of dengue in the entire country.

A key point in controlling dengue spread is the proper monitoring of *A. aegypti* populations, as their increase tend to quickly lead to an increase in dengue transmission [6]. Some possible approaches for estimating *A. aegypti* populations or for detecting population density changes include the use of specialized sensors and processing techniques to identify the mosquitoes and perform some form of counting. In [7], for instance, the authors analyze the sounds emitted by mosquitoes during flight, and develop a full description associated to the *A. aegypti*. An approach based on both mosquito sounds and behaviors is discussed in [8], in which the authors analyze mating behaviors and relate them to tone frequencies; they also mention possible implications to vector control programs. The association between sensing specific mosquito sounds and performing mosquito counts is discussed in [9], and the authors propose a prototype acoustic detector.

However, these solutions have the important limitation of requiring specific sensors that should not be left unprotected in monitoring areas for several days, due to the risk of stealing or tempering with, which could turn the measures unreliable. Even with some works discussing the possibility of low-cost solutions [9–11], the required sensors must be exposed to collect the data, and they are still not commonly used in Brazil, for instance.

The most common solution in Brazil for detecting changes in *A. aegypti* population sizes and estimating densities uses the oviposition traps or ovitraps. These are special traps for collecting the *A. aegypti* eggs [12, 13]. This technique has been shown to be a simple, yet efficient, method for monitoring infested areas of the *A. aegypti* and *Aedes albopictus* mosquitoes.

The use of ovitraps, as a method of entomological surveillance, is considered more economical and operationally feasible than others surveys of larval infestation indices, in emerging and even some developed countries. These traps can produce better measures of risk of being close to the adult *A. aegypti* females, and can also lead to earlier detection of new infestations, as compared to other strategies used today [14].

**Figure 2:**
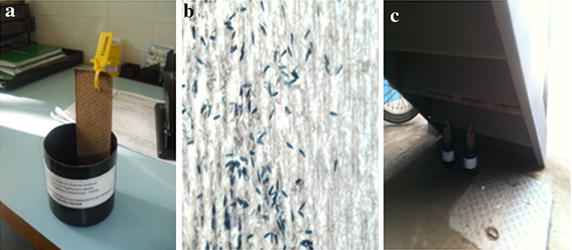
DIVAL-Gama’s Ovitrap (**a**), mosquito eggs on cardboard (**b**) and final installation location (**c**) [21].

The ovitraps generally consist of a black plastic matte bucket, with an opening of 5 cm in diameter by 12 cm in depth, without a lid, with a cardboard made of wood (2 cm × 12.5 cm). The cardboard wrinkled face is facing up to facilitate the adherence of the eggs placed by the female *A. aegypti* mosquito, as Figure 2 shows. There are about 200 ml of water or grass infusion in the bucket, which attracts the female individuals. This cardboard is fixed vertically to the bucket, with or without a paper clip and having most of its size merged into the water. This position allows one to monitor the number of mosquito eggs in order to indicate the presence and level of infestation of mosquitoes in a particular area [14].

In order to allow for a fast evaluation of the number of eggs in such an ovitrap, as opposed to the manual approaches still conducted in Brazil, in our previous work we evaluated some semi-automatic strategies. In [15], for instance, we developed an image processing approach based on artificial neural networks, whose output estimates were compared with the manual counting performed by specialized technicians. In [16], we briefly described how a mobile-ready, different solution could be implemented, based on Java image processing routines.

We also developed a low-cost hardware solution for the acquisition of sequences of images from ovitraps’ cardboards. The solution, first described in [17], uses a specially designed box with a structure that supports the cardboards below a microscopic camera. The camera is moved by microcontrolled motors, in such a way that the technician can automatically collect images from all the positions of the cardboards, with a resolution that allows for eggs counting.

These preliminary systems, however, are limited to the egg counting problem, but still lack the proper Web structure and GIS methods for fast dissemination of information regarding geographical distributions of *A. aegypti* populations. Also, the system in [15] is still not ready for implementation on mobile devices, that can be carried by the technicians in a daily basis to gather the data efficiently an in a timely manner. In fact, it is a MatLab^®^ implementation that operates in an offline manner over a set of previously acquired images. In [16], on the other hand, we introduced the basic idea for a different, Java-ready implementation, that could later be developed on mobile devices for eggs counting.

In this context, in this paper we introduce the complete DIP algorithm to perform the counting of eggs deposited over the ovitraps’ reeds, with integration to a web based SCSA, and with automatic georeferencing of the collected data into a GIS. The system also allows the collected information to be automatically made available on a Web platform. Also, we describe the procedures we performed in order to evaluate the complete system’s performance.

## Methods

The system herein proposed was built based on a field survey. The information has been provided by the professionals working at the Board of Health Surveillance of Brazil’s capital, Brasília (Distrito Federal—DF). In order to take into account all the administrative regions of Brasília, such as Gama, where one of the University of Brasília *campi* is based, a joint venture had to take place. The project in itself had the assistance of the Gama Board of health Surveillance (DIVAL-Gama), who provided 84 cardboards used in 84 ovitraps. These ovitraps were placed in 42 locations in the city of Gama, monitored by DIVAL’s laboratory technicians and Gama UnB researchers. During three days per week, the team collected the cardboards and replaced them with clean ones. The collected cardboards were taken to DIVAL’s lab where the researchers performed manual eggs counts using a microscope. A daily analysis in search for *A. aegypti*’s eggs in the cardboard received the assistance of UnB researchers as to compute the counting with the aid of the SCSA-WEB platform.

DIVAL’s lab technicians work proved to be time-consuming, increasing misinterpretations in manual eggs counting, when more than one technician tried to interpret what they saw through the lens of the microscope. We do understand that the work of such professionals becomes very exhausting and is subject to variations in their readings, throughout the day.

Figure 2 shows an ovitrap in which the cardboard has a yellow label strap with a printed tracking code (a), as well as a zoomed region on this cardboard (b) and an example of ovitrap installation under a residential stair (c).

The software used for the development obeys the GNU GPL compliance. The codes are open and allows for the use and modification of any part off it, free of charge, for the development of our study. The construction of the database used the Workbench 5.2 for data modeling and PostgreSQL. For web application we used Java JSF, CSS, HTML 5 and Jboss web server. As to guarantee the portability of our codes, we have selected the Java programming language using the Juno Eclipse development tool kit for the implementation of the DIP algorithm.

**Figure 3:**
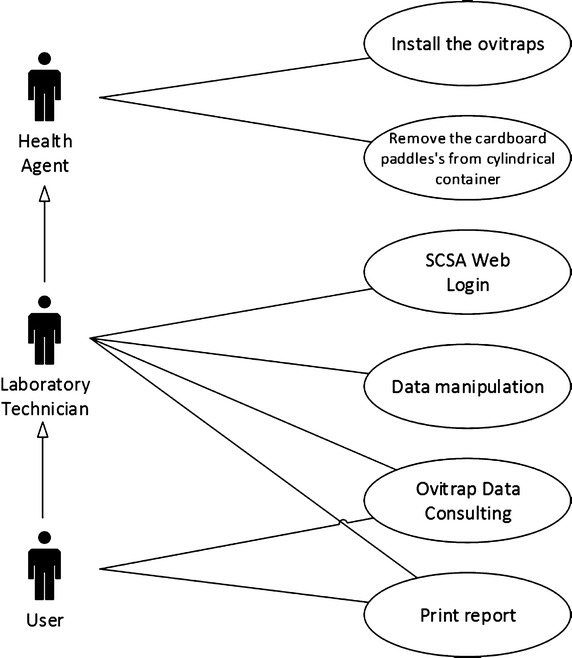
Use case diagram for the SCSA-WEB application.

In Figure 3, a use case diagram illustrates the actions that allows us to perform the counting of the *A. aegypti* eggs. The diagram shows the functions of the following professionals: Health Agent, laboratory technician and end user, i.e. people who interact with the system, represented by actors in black.

The first actor is the Health Agent, who visit homes, shops, and businesses. In the city of Gama, the installation of the ovitraps and cardboard withdrawal are made weekly, and being so it is effective for dengue control in accordance with the Federal District Government health program. All retrieved cardboards are subsequently sent for analysis to the laboratory.

Second actor represents the laboratory technician, who performs the manual eggs counting technique with the aid of a digital microscope. All written information is reported on a regular paper for future reference and analysis.

The third and last actor is a user, known as a health professional, who uses this information to manage dengue fever prevention measures. This only occurs if the user has a profile registered in the system by the administrator.

**Figure 4:**
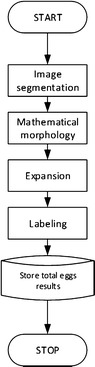
Digital image processing flow chart.

**Figure 5:**
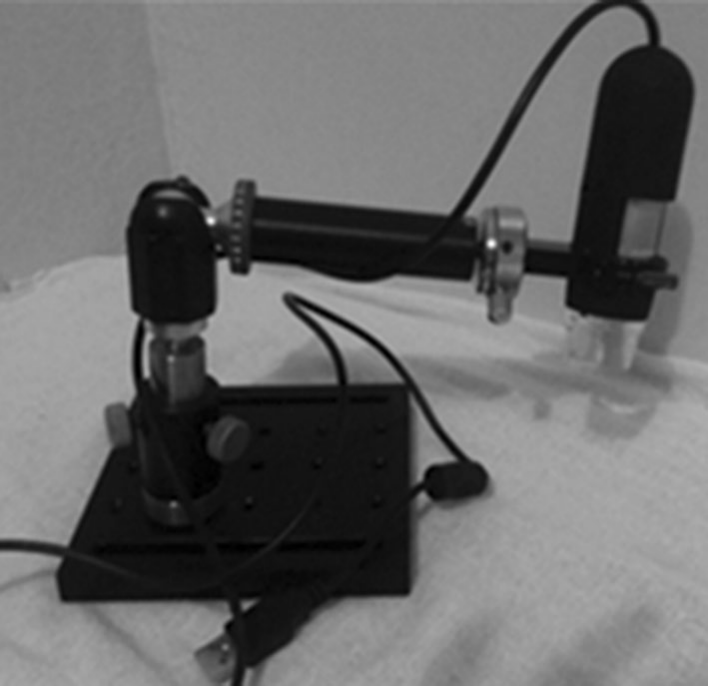
Digital microscope.

In order to create the automated egg counting procedure, the DIP technique used for the construction of the algorithm in Java will initially receive an image sample and then proceed through the following algorithm stages: Image segmentation, mathematical morphology (dilation and erosion) procedures, expansion and labeling, as shown in Figure 4. These techniques are considered suitable for binary images analysis [18]. Before starting the image segmentation, the image must be captured using a digital microscope (Figure 5) with a predefined format, and imported into a computer.

The initial stage of the algorithm uses an image segmentation procedure that reveals distinct partitions of an image correlating with objects or attributes of interest. It is a process of grouping pixels with similar attributes required at the second flow chart stage by the morphological analysis tool.

We use a morphological analysis stage, based on dilation and erosion, for the extraction of imaging components that are of interest for the representation and description of regions, such as edges and areas, among others. The dilation is a technique that decreases and increases the image size [18]. Erosion removes pixels of solid areas in an image, i.e., the purpose of erosion is to remove unwanted pixels in certain regions.

For the construction of the GIS, we chose QuantumGIS and I3Geo for shapesfiles maps editing and manipulation, as well as PostgreSQL as the DBMS, as it is robust and appropriate for geographical databases.

After applying both DIP and GIS tools, the egg counts are saved in the DB, uploaded and georeferenced into a web server platform. This approach provides us with analysis and support tools to combat the dengue fever, based on information made available in a timely manner, in order to inform one about occurrence rates and to generate outbreaks alerts.

## Results

**Figure 6:**
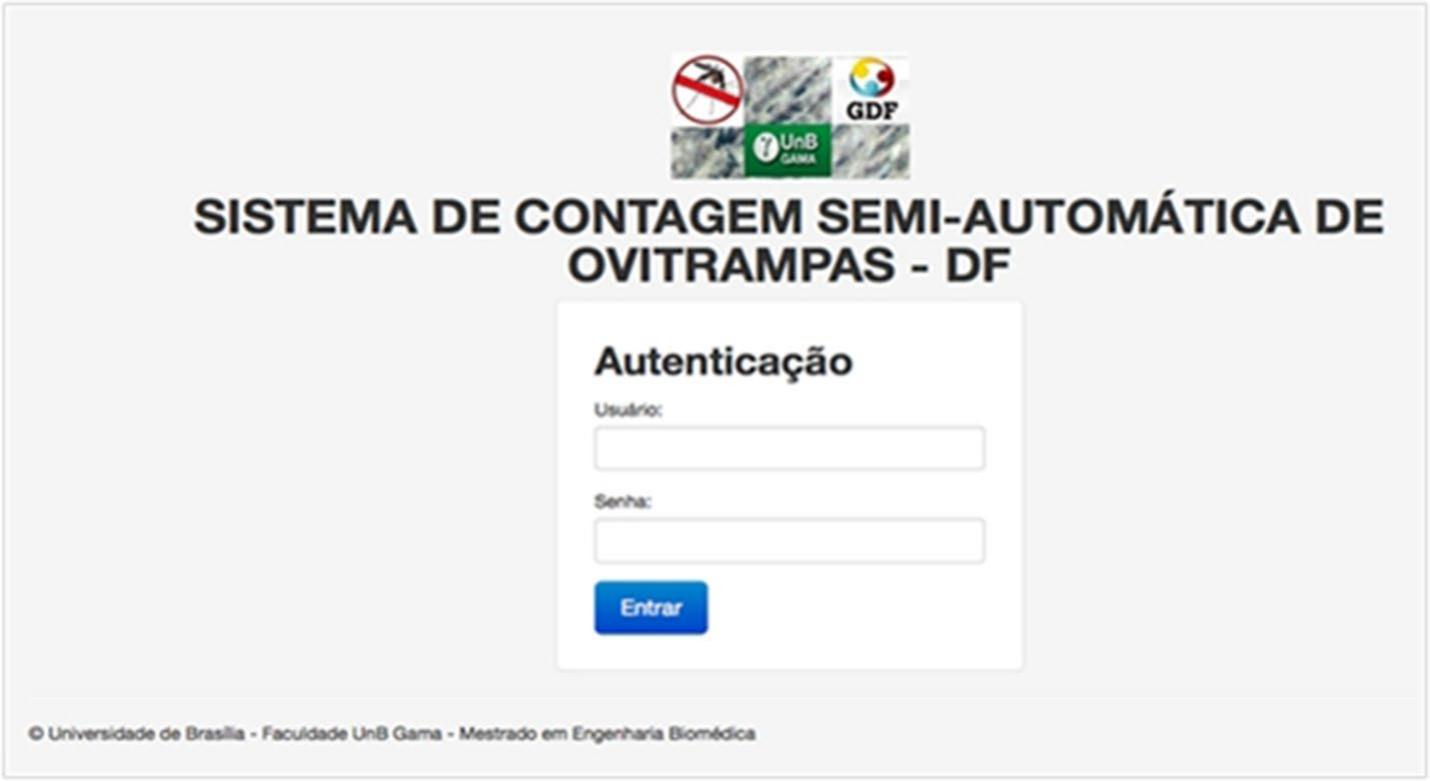
SCSA-WEB authentication screen.

As initial proof of concept, high definition picture samples of the cardboards were taken, identified and stored. Any of the actors shown in Figure 3 has access to the SCSA-WEB, using a login and password, as shown in Figure 6. All actors activities are audited and sent to the PostgreSQL.

**Figure 7:**
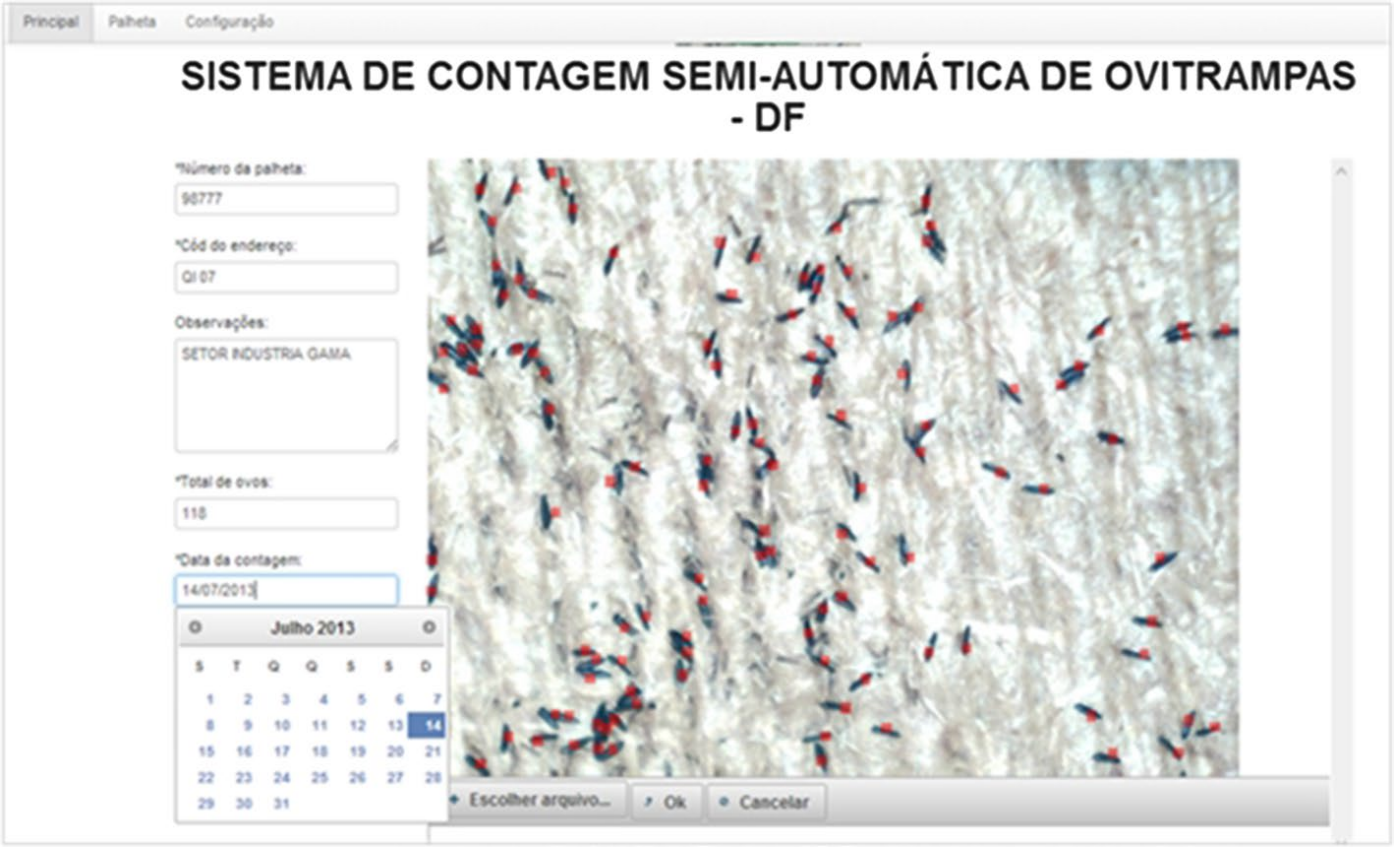
Egg count screen with mouse clicks in *red*.

Figure 7 shows our new semi-automatic egg count system, known as SCSA-WEB platform. The user can choose any image from a given directory containing the picture samples and write any information at the input fields shown on the left of the screen. The egg count is initialized using a left mouse button click, and the semi-automated counting can then be observed, on red dots. The counting result is automatically updated on each mouse click and presented at the output field shown on the left of the screen. SCSA-WEB also allows one to generate special reports of the data stored in the DB for further analysis. As stated before, the manual counting by the laboratory technician is time-consuming and prone to error, as he has to maintain his eyes on a microscope counting several eggs at once. Loss of attention would cause new readings and frustrations. Our test results shows that the time used for egg count using our semi-automated SCSA-WEB has being considerably reduced as there is now an image showing eggs that have been already counted.

In practice, the SCSA-WEB program is meant to receive any image size and resolution (in jpeg or png format) that can be held by the DIP algorithm in its automated egg count.

**Figure 8:**
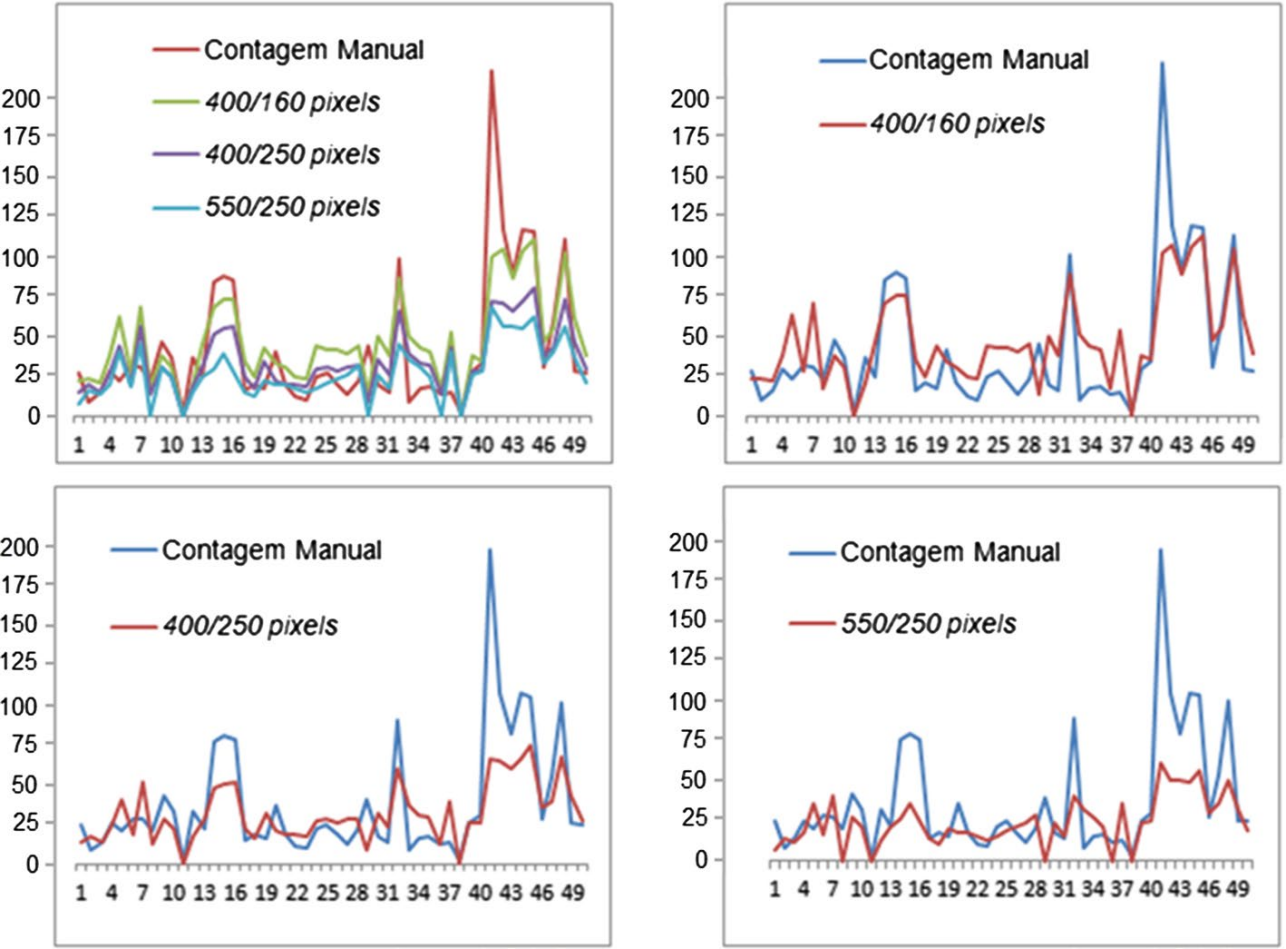
Comparison of manual and automated egg count over 50 pictures samples.

The first chart shown on the left size of Figure 8 provides a comparison of the results obtained by manual and automated egg counts. The 2nd, 3rd and 4th lines represent the automatic count made in Java and the respective pixels adjustments with their respective calibration parameters. On the horizontal line there is a total of 50 picture samples. The first red line represents the manual count, the second green line represents a 400 × 160 pixels image, the 3rd purple line a 400 × 250 image and the fourth blue line a 550 × 250 image.

The 2nd, 3rd and 4th charts represent manual (in blue) and automated Java (in red) egg counts, with a 400 × 160, a 400 × 160, and a 400 × 160 image, respectively.

At first, there is a clear evidence of discrepancies between samples values 5, 7, 15, 16, 29, 33, 41, 42, 43, 44 and 48, regarding the observed egg counts. The difference in values can be explained by the following reasons: the current cardboards samples (wet, dry and/or bending) and microscope adjustments (focus and height configurations) used at that time. Otherwise, the automated egg counts using different pixels size are very close to the results obtained using manual egg counts. But, we still have to find which image configuration is best suited for a human manual count replacement.

Table 1 shows a statistical comparison of egg count results. We used the mean, standard deviation, minimum value, maximum value and Wilcoxon’s P value for better data analysis [19, 20].

**Table 1:** Description of the statistical values of egg count

	Manual count	400 ×160 pixels	400 × 250 pixels	550 × 250 pixels
Mean	27.00	40.50	30.00	24.00
Standard deviation	40.57	26.92	19.40	16.50
Minimum value	1	0	0	0
Maximum value	214	110	80	67
P value		0.015	0.215	0.006

Applying the Wilcoxon test, for values of ${P > 0.05}$, it accepts the null hypothesis (H0) [19, 20], i.e. there is no significant difference between the total eggs observed by manual count and the ones estimated by the DIP Java algorithm. Otherwise, for ${P < 0.05}$, the estimates are significantly different.

According to Table 1, it can be observed that the results obtained by the 400 × 250 images reflect the best approximation when compared to the manual count. For all other comparisons, the differences are statistically significant (P value less than 0.05).

**Figure 9:**
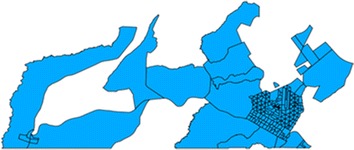
The city of Gama’s Shapefile displayed on QuantumGIS.

Now, knowing the appropriate image size so that the proposed DIP technique matches the manual counts, all data obtained through automation can be stored in the DB. As to geographically display and analyze the stored data, we have implemented a GIS Web interface. First, we had to manipulate and store Gama’s Shapefiles, through the Quantum GIS application, shown in Figure 9, also known as QGIS (A Free and Open Source Geographic Information System). This application accepts shapefiles that can be edited and manipulated.

**Figure 10:**
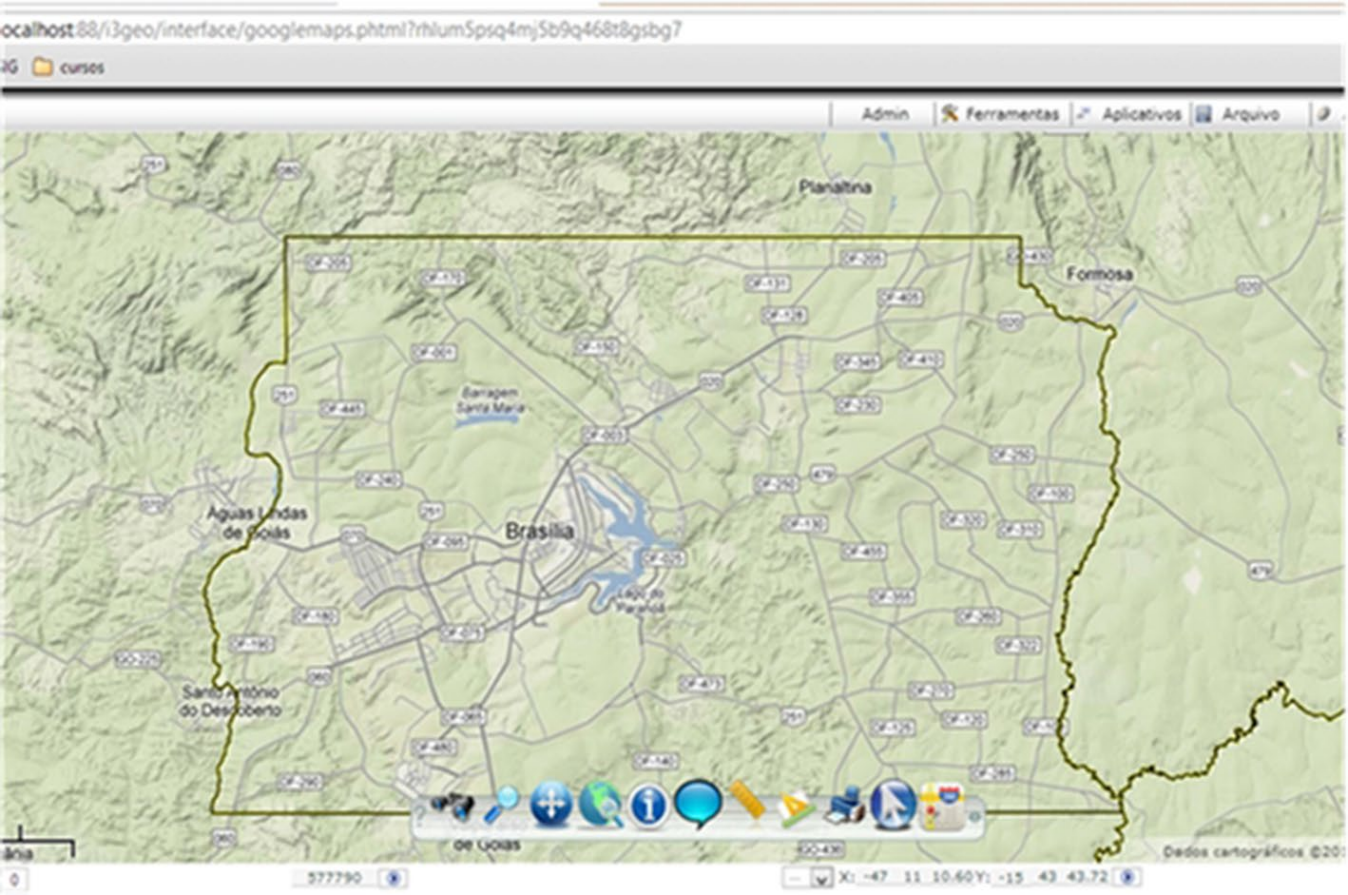
I3Geo intranet Web platform centered in Brasília, before deployment.

Next step was to transfer the shapefiles to I3Geo. Figure 10 shows our intranet Web platform displaying Brasília and Gama’s shapefile on I3Geo. The Web platform is linked to a DBMS which in turn receives the automated DIP egg count values collected in the region.

Both QGIS and I3Geo serve as an analysis and support tools to combat dengue fever in the Federal District, providing the information in a timely manner as it is done automatically with a single DBMS. The new GIS Web Platform is called SIGO-Dengue. We believe that the Environmental Surveillance Directory (DIVAL-Gama) and Brasília’s Board of Health (SSDF), in partnership with UnB, can benefit from this scenario in a near future.

## Discussion

A management structure and procedures for maintenance of the SIGO-Dengue Web platform are being discussed, as well as periodic backup routines. Still, the importance of an easy access to the system, as well as training human resources for correct handling of the platform and seeking improvements to the healthcare industry, should be considered.

The SCSA-WEB was built based on field research carried out in about three months. The information has been provided by healthcare professionals and technicians working at the Secretariat of Health surveillance of DF. The screens can be improved and changed according to the need and demand of any healthcare professional. All technological resources must be improved in order to increase the overall system performance.

**Figure 11:**
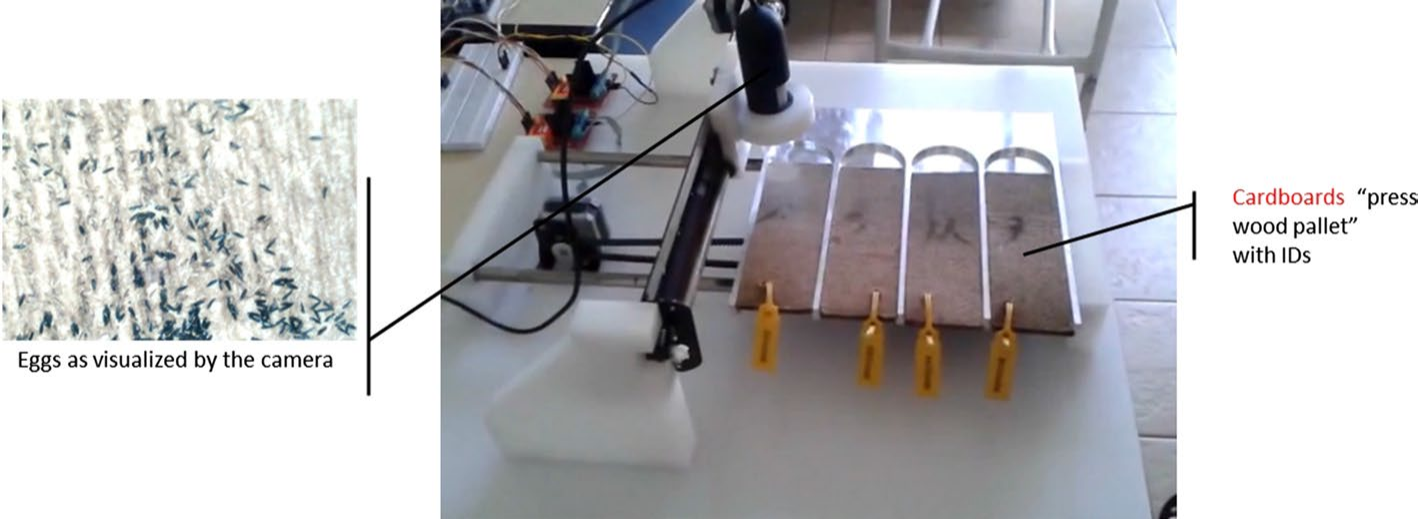
Electromechanical system made at LIS [22, 23].

The DIP system developed in Java is now being improved, particularly with respect to filtering techniques, egg counting, and the possibility of processing more than one cardboard for automated digital microscope readings. Figure 11 shows a novel, but still in test, platform that would have four cardboards, a digital microscope with horizontal and vertical movement displacements and a digital on-board technology system running the DIP Java algorithm.

We understand that despite all preventive measures and advances in Information Technology (IT), strategies for dengue control seems insufficient, because thousands of people continue to be affected by the disease in the world. So, it becomes necessary to have a continuity in scientific projects and technologies development. The society has to learn and understand daily prevention techniques. Therefore, the proposal of an automated egg count program aims to improve the deployment of the SIGO-Dengue project in research of ovitraps automation and wirelessly tracing endemic areas against the spread of dengue fever. The use of technological resources as a tool to generate quick and reliable information is of extreme importance, in order to support government decision-making to prevent other victims of the disease in Brazil.

## Conclusion

We propose, implement, and evaluate an automatic system for counting the eggs that *A. aegypti* individuals deposit on the cardboard of ovitraps, combined with a geographic information system (GIS) and an automated Web platform for fast communication of collected data. These resources allow the definition of risk levels of dengue transmission, based on the estimation of local populations of the transmitting mosquito. They also allow the prompt dissemination of this information, in order to guide public health measures and to enhance population awareness of risks.

The system can also improve SSDF’s health surveillance services based on the developed IT tools. Both SCSA-WEB and DIP serve to streamline the work of health workers assisting in the detection of any Dengue outbreak.

The development of the SCSA-WEB application is an important addition, as it is used as a reference for conducting the eggs counting with a simple user interface and for comparing the results with the automatic counting, using the DIP algorithm program developed in Java. The SCSA-WEB has resources that can be use in daily life by health technicians. Instead of filling all information manually, they now have the possibility of using an automated system with more responsive commands and of generating file reports with ease.

The new DIP algorithm confirms the estimate and efficiency of performing an automatic egg count. The automated egg count technique approaches the manual count values, thus validating our Java program implementation.

Finally, note that the low-cost ovitraps combined with the developed SCSA-WEB and DIP systems make the proposed platform useful also for analysing potential endemic risks other than dengue. In fact, diseases such as malaria, the *Trypanosoma cruzi* disease, yellow fever, and others all depend on transmitting vectors that need a fixed places to reproduce and grow. As future work, we will test our proposed system in evaluating endemic areas affected by these other diseases, and will complete the integration between the data acquisition system with the DIP and SCSA-WEB algorithms.
